# An Effective Critical Care Unit Admission Policy for Patients With Diphenhydramine Overdose: A Case Report

**DOI:** 10.7759/cureus.100578

**Published:** 2026-01-01

**Authors:** Yojiro Kashimura, Ayane Sato, Satoshi Toriumi, Mitsumasa Tsuchiya, Koichi Ueno

**Affiliations:** 1 Department of Emergency and Critical Care Medicine, Kawasaki Municipal Hospital, Kawasaki, JPN; 2 Deparment of Neurology and Neurological Endovascular Therapy, Kawasaki Municipal Hospital, Kawasaki, JPN

**Keywords:** critical care unite, dphenhidramin overdose, latent long qt syndrome, rescusitation, torsade de pointes

## Abstract

Although many patients who overdose on over-the-counter (OTC) medications are brought to the emergency and critical care departments, determining the need for hospitalization remains challenging. In particular, uncommon diseases such as congenital long QT syndrome (CLQTS) are often difficult to detect in the emergency department. On the other hand, overlooking this condition can be critical, because it causes fatal arrhythmias. Therefore, clinicians should be aware of its seriousness.

A 24-year-old female was brought to our hospital after consuming alcohol and ingesting ten 40 mg tablets of diphenhydramine. She had a history of an electrocardiographic abnormality identified during a routine medical check-up. On arrival, she complained of nausea. She was 155 cm tall and weighed 45 kg, and her vital signs were stable. After infusion of 500 cc of acetated Ringer solution with 5% dextrose, her nausea improved, and she denied suicidal intent and expressed a desire to return home. However, following careful discussion, she was admitted to the emergency critical care unit because her QTc was prolonged to 480 msec, and she had a prior history of ECG abnormalities noted during routine screening. Two hours after arrival, she suddenly developed torsade de pointes (TdP), and cardiopulmonary resuscitation was initiated immediately. The arrhythmia terminated within one minute but recurred 15 minutes later. Cardiopulmonary resuscitation was reinitiated, followed by unsynchronized cardioversion, which successfully resolved the episode. Afterward, she experienced no further cardiac events and was discharged on the third day after admission.

Latent long QT syndrome was identified during outpatient follow-up two months after discharge. Although this single case does not allow us to conclude that all patients with diphenhydramine overdose accompanied by QTc prolongation and a history of cardiac abnormalities should be hospitalized, our admission policy appeared to be appropriate in this instance. Further evaluation through additional cases or an observational study may be necessary to establish clear hospitalization criteria for diphenhydramine overdose.

## Introduction

Many patients with over-the-counter (OTC) medication overdoses are brought to emergency and critical care departments. However, clear criteria for hospitalization in these patients are lacking. Moreover, uncommon conditions such as congenital long QT syndrome (CLQTS), which can potentiate the effects of these medications, are often difficult to detect in the emergency department [1-4]. CLQTS is a serious condition, and if it is overlooked, it may lead to fatal arrhythmias such as pulseless ventricular tachycardia (VT) and torsade de pointes (TdP), resulting in sudden death. Therefore, clinicians should recognize its clinical significance. Nevertheless, determining the indication for hospitalization in patients who have overdosed on QTc-prolonging medications remains challenging [1-4].

In 1997, Zareba et al. first reported ECG changes induced by diphenhydramine, and several cases of torsade de pointes caused by over-the-counter medications have since been reported [5-10]. However, a definitive method for determining hospitalization in the emergency and critical care department has not been established. Therefore, defining clear indications for hospitalization in cases of diphenhydramine-induced TdP is an urgent issue. In this report, we describe a patient with a diphenhydramine overdose who developed TdP. The patient was admitted to the critical care unit and was successfully managed with prompt critical care.

## Case presentation

A 24-year-old woman with a history of bipolar disorder was brought to our department following alcohol consumption and an overdose of over-the-counter medications. She had ingested two cans of highball and ten 40 mg tablets of diphenhydramine for recreational purposes. She also had a history of electrocardiographic abnormalities detected during a routine health check-up, although no specific diagnosis had been established. There was no family history of cardiac disease. On arrival, she complained of nausea. Her height was 155 cm, and her weight was 45 kg. Vital signs were as follows: Glasgow Coma Scale (GCS) score: E4V5M6, body temperature: 38.0 ℃, blood pressure: 131/80 mmHg, heart rate: 105 beats per minute, and respiration rate: 30 breaths per minute. Cardiovascular examination revealed a normal apical impulse, normal S1 and S2, and no murmurs, rubs, or gallops. There was no peripheral edema, and pulses were symmetric. Laboratory tests (Table 1) revealed a white blood cell count of 13,900μL, hemoglobin of 10.6 g/dL, lactate of 4.4 mmol/L, and C-reactive protein of 0.07 mg/dL.

**Table 1:** Initial laboratory data ^*^Abnormal values WBC: white blood cells; RBC: red blood cells; Hb: hemoglobin; APTT: activated partial thromboplastin time

Test	Result	Reference range
WBC (/μL ×10^3^)	13.9^*^	3.5-9.0
RBC (/μL ×10^3^)	414	350-555
Hb (g/dL)	10.6^*^	13-16
Platlets (/μL ×10^4^)	46.6^*^	15-40
APTT (sec)	20.0^*^	24.0-39.0
Fibrinogen (mg/dL)	274.9	200-400
Total protein (g/dL)	7.1	6.2-7.5
Albumin (g/dL)	3.5	3.0-3.6
Total bilirubin (mg/dL)	0.7	0.4-1.5
Aspartate aminotransferase (U/L)	28	13-30
Alanine transaminase (U/L)	19	13-23
Blood urea nitrogen (U/L)	8	7.5-17.5
Creatinine (mg/dL)	0.62	0.5-1.8
Sodium (mmol/L)	140	136-147
Chlorine (mmol/L)	106	102-108
Potassium (mmol/L)	4	3.4-4.7
Calcium (mg/dL)	8.5	8.2-10.0
Phosphate (mg/dL)	3.1	2.5-4.5
Magnesium (mg/dL)	2.1	1.5-2.2
Glucose (mg/dL)	97	73-109
Lactate (mmol)	4.4	0.0-2.0
C-reactive protein (mg/dL)	0.07	＜0.3

**Figure 1:**
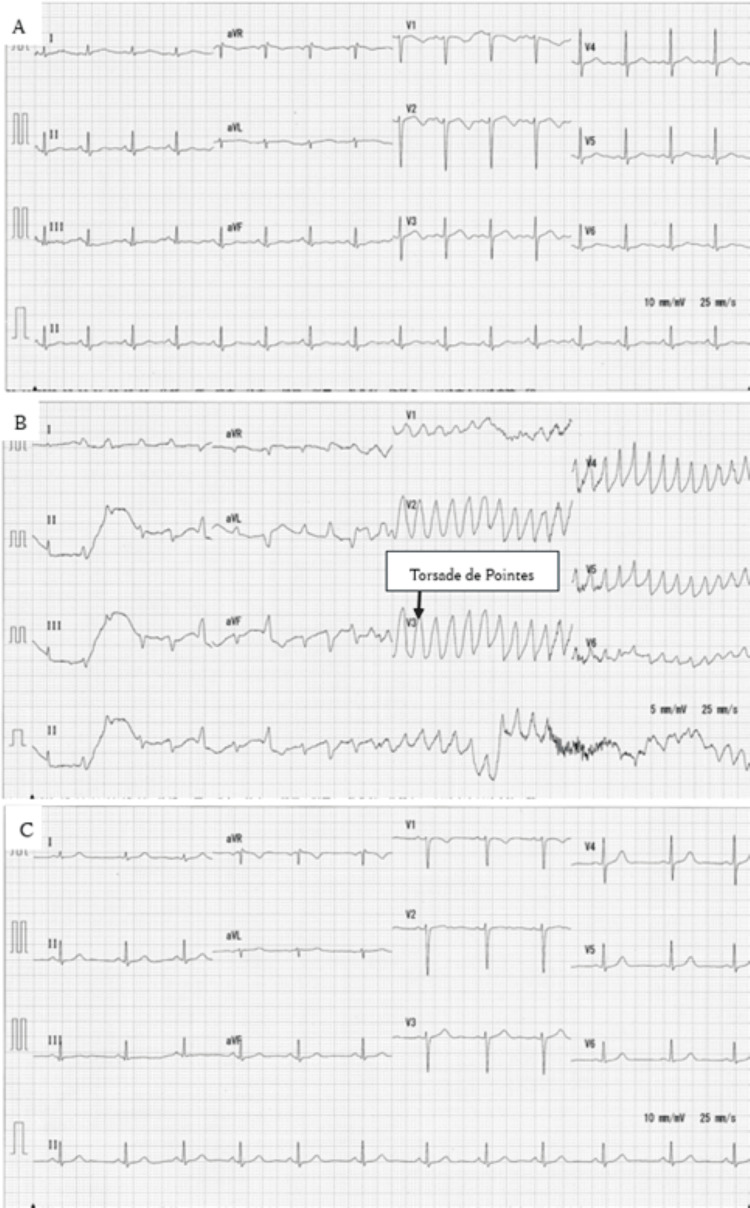
Timeline of electrocardiogram findings A: Electrocardiogram on arrival demonstrating QTc prolongation. B: Electrocardiogram demonstrating the Torsade de Pointes, which is shown by the arrow. C: Electrocardiogram demonstrating normal findings

Her ECG (Figure 1) showed a prolonged QTc interval of 480 msec. After infusion of 500 cc of acetated Ringer solution with 5% dextrose, her nausea improved. She denied any suicidal ideation and expressed a desire to go home. Following careful discussion, she was admitted to the critical care unit because of the prolonged QTc interval and a history of ECG abnormalities detected during a routine check-up. Two hours after the overdose, she suddenly developed pulseless VT consistent with TdP on continuous monitoring, and cardiopulmonary resuscitation was immediately initiated. The arrhythmia resolved within one minute but recurred 15 minutes later (Figure 1). Cardiopulmonary resuscitation was reinitiated, and unsynchronized cardioversion was performed, successfully resolving the TdP. Intravenous magnesium sulfate was administered, and a follow-up ECG showed a QTc interval of 439 msec (Figure 1).

A cardiology consultation was requested, and continuous 12-lead ECG monitoring was initiated. The patient experienced no further cardiac events after the episode and was discharged three days later. An ECG performed 20 days after discharge showed no abnormalities, and she was referred to another hospital due to relocation. Two months later, she was diagnosed with latent long QT syndrome at that hospital.

## Discussion

Rephrase. No em dashes: Diphenhydramine-induced ECG findings were first reported in 1997, followed by a report by Radovanovic et al on the dose-dependent toxicity of diphenhydramine overdose. Since then, several cases of diphenhydramine-induced TdP have been reported. While these reports indicated that diphenhydramine induces QTc prolongation and sometimes provokes TdP, the ECG abnormalities commonly appear when the overdose is above 1.3 g, and the past report demonstrated that the lethal dose of diphenhydramine is 40mg/kg [5-10].

Furthermore, critical TdP can be triggered by a combination of congenital and acquired factors, such as diphenhydramine overdose in a patient with congenital long QT syndrome (CLQTS). Diagnosing CLQTS is challenging because a definitive diagnosis requires a Holter ECG or an epinephrine stimulation test. Importantly, one-third of CLQTS patients present with borderline QTc intervals, and approximately 12% have a QTc under 440 msec [3-4]. In theory, outpatient follow-up may be appropriate if the diphenhydramine dose is below the lethal threshold and there is no family history of cardiac disease, since there is no reliable method to identify latent long QT syndrome. The central question is whether all patients with diphenhydramine intoxication require hospitalization. In this case, we hesitated to admit the patient because her nausea had improved and she wished to return home.

Diphenhydramine-induced TdP has not been supported by robust evidence, and the optimal management of diphenhydramine overdose remains unclear. In our case, we decided to admit the patient because she had a history of ECG abnormalities and a prolonged QTc interval. Following this policy, the patient was hospitalized, and she subsequently developed TdP after the diphenhydramine overdose. Although this single case does not allow us to definitively confirm the appropriateness of our admission policy, it suggests that hospitalization was reasonable in this situation. Nevertheless, precise criteria for admitting all patients with diphenhydramine overdose remain undefined, and further evaluation is required.

## Conclusions

In this case, we established an admission policy for patients with diphenhydramine overdose based on at least one of the following criteria: 1) a family history of cardiac events, 2) a personal history of cardiac events or ECG abnormalities, or 3) a current QTc abnormality. The patient was hospitalized because her QTc was prolonged and she had a history of ECG abnormalities identified during a routine medical check-up. She subsequently developed TdP after admission. Reports of diphenhydramine-induced TdP are too few to definitively establish admission criteria. Therefore, a cautious admission policy may be appropriate at this time. Although our admission policy was suitable in this case, we cannot conclude that it is always correct or the best approach for all patients with diphenhydramine overdose based on a single case. The optimal criteria for hospitalization remain uncertain, and further evaluation through additional cases or an observational study is needed to establish clear guidelines.
